# Mycorrhizal Fungi Respond to Resource Inequality by Moving Phosphorus from Rich to Poor Patches across Networks

**DOI:** 10.1016/j.cub.2019.04.061

**Published:** 2019-06-17

**Authors:** Matthew D. Whiteside, Gijsbert D.A. Werner, Victor E.A. Caldas, Anouk van’t Padje, Simon E. Dupin, Bram Elbers, Milenka Bakker, Gregory A.K. Wyatt, Malin Klein, Mark A. Hink, Marten Postma, Bapu Vaitla, Ronald Noë, Thomas S. Shimizu, Stuart A. West, E. Toby Kiers

**Affiliations:** 1 Department of Ecological Science, Vrije Universiteit, De Boelelaan 108, 1081 HV Amsterdam, the Netherlands; 2 Department of Zoology, University of Oxford, 11a Mansfield Road, Oxford OX1 3SZ, UK; 3 Balliol College, University of Oxford, Oxford OX1 3BJ, UK; 4 AMOLF Institute, Science Park 104, 1098 XG Amsterdam, the Netherlands; 5 Section of Molecular Cytology and van Leeuwenhoek Centre for Advanced Microscopy, Swammerdam Institute for Life Sciences, University of Amsterdam, 1098 XH Amsterdam, the Netherlands; 6 Harvard T.H. Chan School of Public Health, 677 Huntington Avenue, Boston, MA 02115, USA; 7 Faculté Psychologie, Université de Strasbourg, 12 Rue Goethe, 67000 Strasbourg, France

**Keywords:** biological markets, symbiosis, hoarding, mutualism, arbuscular mycorrhizae, conflict, cooperation, quantum dots, economics, inequality

## Abstract

The world’s ecosystems are characterized by an unequal distribution of resources [1]. Trade partnerships between organisms of different species—mutualisms—can help individuals cope with such resource inequality [2, 3, 4]. Trade allows individuals to exchange commodities they can provide at low cost for resources that are otherwise impossible or more difficult to access [5, 6]. However, as resources become increasingly patchy in time or space, it is unknown how organisms alter their trading strategies [7, 8]. Here, we show how a symbiotic fungus mediates trade with a host root in response to different levels of resource inequality across its network. We developed a quantum-dot-tracking technique to quantify phosphorus-trading strategies of arbuscular mycorrhizal fungi simultaneously exposed to rich and poor resource patches. By following fluorescent nanoparticles of different colors across fungal networks, we determined where phosphorus was hoarded, relocated, and transferred to plant hosts. We found that increasing exposure to inequality stimulated trade. Fungi responded to high resource variation by (1) increasing the total amount of phosphorus distributed to host roots, (2) decreasing allocation to storage, and (3) differentially moving resources within the network from rich to poor patches. Using single-particle tracking and high-resolution video, we show how dynamic resource movement may help the fungus capitalize on value differences across the trade network, physically moving resources to areas of high demand to gain better returns. Such translocation strategies can help symbiotic organisms cope with exposure to resource inequality.

## Highlights

• Quantum-dot technology can be used to track trading strategies of mycorrhizal fungi
• Increasing exposure to inequality stimulates trade and resource movement
• Fungal trade strategies are not uniform across the symbiotic network
• Fungi can capitalize on trade by first moving resources to areas of high demand

## Graphical Abstract

**Figure:**
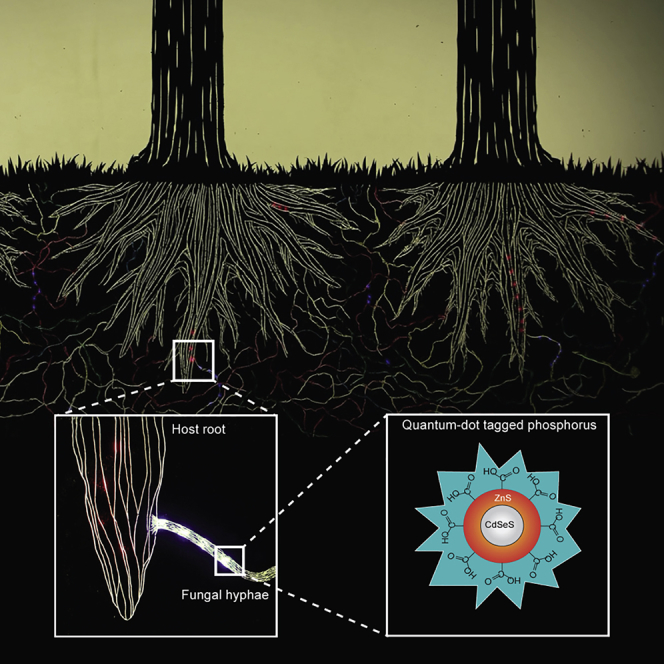

## Teaser

Inequality can shape trade patterns in nature. Whiteside et al. develop a quantum-dot technique to track trade strategies of mycorrhizal fungi exposed to resource inequality across their networks. They find inequality stimulated trade, with fungi exploiting potential gains from host plants by moving resources from rich to poor patches.

## Results and Discussion

Mutualistic partnerships are ubiquitous [4] and allow species to colonize diverse environments that fluctuate dramatically in resource availability, from our mammalian guts [9] to deep-sea trenches [10]. Although mutualistic trade can help individuals, the relative benefits to each partner will shift according to how resources are distributed [2, 3]. As resources become increasingly patchy in time or space, returns can become more variable, and thus less reliable [7, 11, 12]. Consequently, individuals may be favored to hoard resources—be it for consumption, to retain a competitive edge, or for trading later [6, 13, 14]. This can lead to a decrease in current trade. Alternatively, individuals may be able to exploit local resource variation to dictate favorable terms of trade [2, 7]. These higher returns would lead them to invest more heavily in trade. However, because of our inability to precisely track how resources are moved between different species, it has not been possible to test these hypotheses about how exposure to resource inequality influences trading strategies in mutualisms.

We developed a quantum-dot nutrient-tracking technique that allowed us to track the trade of fluorescently labeled phosphorus in arguably the world’s most widespread trade partnership: the mutualism between arbuscular mycorrhizal fungi and land plants [15]. Arbuscular mycorrhizal fungi form underground networks of filamentous hyphae in the soil [16]. The fungus mobilizes and collects phosphorus from the soil and trades this commodity with its host plants for carbon in a market-like exchange [6, 8, 17, 18, 19, 20]. By tagging phosphorus with highly fluorescent nanoparticles of different colors, we could follow the movement of resources from their points of origin, across a fungus, and into the host root.

**Figure 1:**
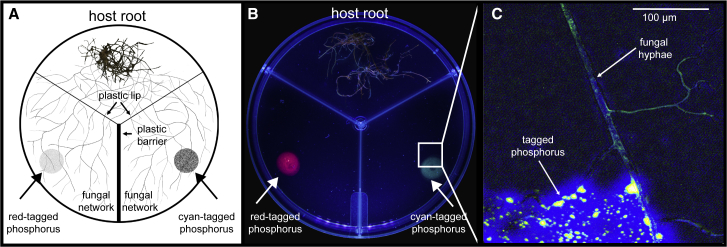
Experimental Design (A) Illustration of the experimental setup in which quantum-dot-tagged phosphorus was added to two nutrient compartments, keeping the absolute amount consistent but varying the ratios to create high (90:10), medium (70:30), and no resource inequality (50:50) across the fungus. Roots were confined to the root compartment, but the fungus was able to cross the plastic lip and enter fungus-only nutrient compartments. A plastic barrier prevented any non-fungal movement of injected nutrients. (B) Mock-up of inequality replicate with resource patches of cyan and red quantum-dot-tagged phosphorus and fungal network. (C) A close-up of a single fungal hypha with quantum-dot-tagged phosphorus in its network. See bar for scale. See also Figures S1 and S2.

Our aim was to examine how the trading strategy of a fungus responds to varying levels of resource inequality. We manipulated resource distributions across a fungus, simultaneously exposing it to rich and poor patches of tagged phosphorus across its network (Figure 1). We then asked whether the fungus responds to higher resource inequality by increasing trade with the host plant, or by hoarding resources and trading less. Because our phosphorus was fluorescently labeled according to whether it came from a resource-rich or -poor patch (Figure S1), we could determine where these phosphorus resources were hoarded, relocated, or transferred to the host.

We varied resource inequality across a fungus colonizing a three-compartment Petri dish (Figure 1A). In one compartment, we grew a host of transformed carrot root (*Daucus carota*). This *in vitro* root organ culture was inoculated with the fungus *Rhizophagus irregularis* and grown under standard conditions [17, 21, 22]. The fungal network, but not the root, was then allowed to cross into two separate fungus-only compartments, which were physically separated by a plastic barrier (Figures 1A and 1B). This allowed nutrients to move within the fungal network and across the root + fungus compartment but prevented nutrients from diffusing between the three compartments.

We varied the level of inequality across the fungus by adding the same overall amount of phosphorus, but by differentially distributing it between the two fungus-only compartments. We injected phosphorus of different colors in a ratio of 90:10, 70:30, or 50:50 between the two compartments, leading to high, medium, and no resource inequality, respectively. To verify there was no color preference in the uptake of quantum dots (Figures S2A–S2C), to test for any toxic side effects (Figure S2D), and to confirm that tagged nutrients accumulated in plant and fungal tissue as expected (Figures S1D–S1F), we simultaneously ran a series of methodological control experiments in both whole plants and *in vitro* root organ cultures.

### Fungal Trading Strategy Depends on Exposure to Inequality

**Figure 2:**
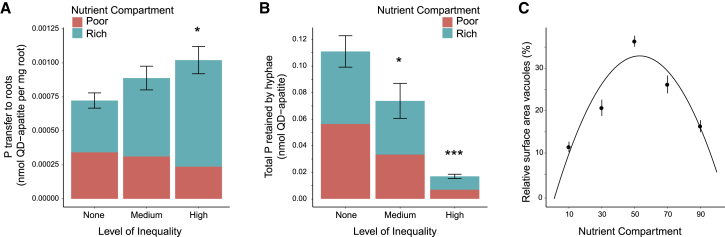
Fungal Trading Strategy (A) Total transfer of phosphorus from the fungal network to the host root is affected by inequality treatment (F_2,72_ = 3.35, p = 0.04, n = 75). Colors indicate the nutrient source compartment with rich = 90, 70, 50 (cyan) and poor = 10, 30, 50 (red), with nmol phosphorus:quantum dot of 708:1. (B) Retention of quantum dot-apatite per total hyphae (F_2,56_ = 37.51, p < 0.01, n = 59) and per mg hyphae (F_2,56_ = 15.42, p < 0.01, n = 59; not shown) is affected by inequality treatment. (C) Relative surface area of vacuoles (nutrient storage structures) in fungal hyphae per nutrient compartment is affected by inequality treatment (χ^2^ = 116.04, degree of freedom [df] = 2, p < 0.01, n = 75 plates), with the highest storage found when there was no inequality. All figures show total means ± SE. Asterisks indicate significant difference compared to no inequality treatment at ^∗^p = 0.05 and ^∗∗^p = 0.01. See also Figures S2 and S3 and Table S1.

We found that inequality had a significant effect on how much phosphorus the fungus traded with the host (Figure 2A). After 60 days of exposure to resource inequality, we harvested the roots and fungal network from our *in vitro* experiment (Figure 1A) and quantified fluorescence (Figure S1G). We found that total transfer of phosphorus by the fungus to the host root increased under exposure to inequality, with the 90:10 inequality treatment showing the highest level of phosphorus transfer per mg of host root (Figure 2A). Transfer was higher when the fungus was exposed to the highest level of inequality, even though access to the absolute amount of phosphorus was the same across treatments, and there were no statistical differences in host root biomass (Figure S3A), intra-radical root colonization (Figure S3B), or total fungal biomass (Figure S3C). This suggests that changing the relative distribution of nutrients across the fungus can change the overall fungal trading strategy, with exposure to high resource inequality leading to increased phosphorus transfer.

We then compared the amount of nutrients transferred to the host by the fungus, according to the nutrient patches the fungus could access. As expected, more of the phosphorus in the host root originated from the phosphorus-rich patch (Figure 2A). However, the relative contributions to the host root from the fungus colonizing different nutrient patches did not match the relative phosphorus distributions—the section of fungus growing in the phosphorus-poor compartment transferred more phosphorus to the host relative to the resources it could access (Table S1). This demonstrates that fungal trade strategies are not uniform across the network: the fungal section in the poor patch over-contributed to trade given the resources it could access, whereas the rich patch under-contributed.

Consistent with the influence of higher inequality leading to more trade, we also found that a fungus exposed to less inequality hoarded more phosphorus in storage structures across its network (Figure 2B). We illuminated regions of the fungus with a laser beam and used raster image correlation spectroscopy to measure the number of tagged phosphate particles in a known biovolume across different inequality treatments (Figure S1F). This spectroscopy technique allowed us to quantify the volume of specific structures within the fungus and study storage patterns across space. We found that a fungus exposed to lower inequality retains more phosphorus (both per total hyphae and per mg hyphae) (Figure 2B), and that it contains larger nutrient storage structures (Figure 2C). This suggests that a fungus is more likely to stockpile resources when it experiences low inequality across its network.

### Fungi Translocate Nutrients from Resource-Rich to Resource-Poor Patches

A fungal network can alter phosphorus trade with hosts by changing the amount of phosphorus it trades (Figure 2A), but also by altering where across the network it transfers that phosphorus [16, 19, 23]. In nature, arbuscular mycorrhizal networks can be meters long and simultaneously exposed to radically different nutrient conditions. The hyphae of these fungal networks lack dividing walls (aseptate) and so are open and continuous, which allows for resources to be moved across their networks [24]. If external resource conditions vary spatially (e.g., [25]), there is a potential for the fungus to gain different returns from the symbiosis by trading phosphorus at different locations of the network [19]. This would favor a redistribution of resources within a network, before trade. Consequently, we asked whether exposure to inequality affected the degree of nutrient movement across the fungus. Specifically, we asked whether a fungus moves phosphorus away from rich patches, where it is potentially in low demand, to poor patches of the network, where resource demand from the host—and the fungus itself [26]—is potentially higher. Because we had tagged our phosphorus resources with nanoparticles of different colors depending on their source of origin, we could track their distribution within the network.

**Figure 3:**
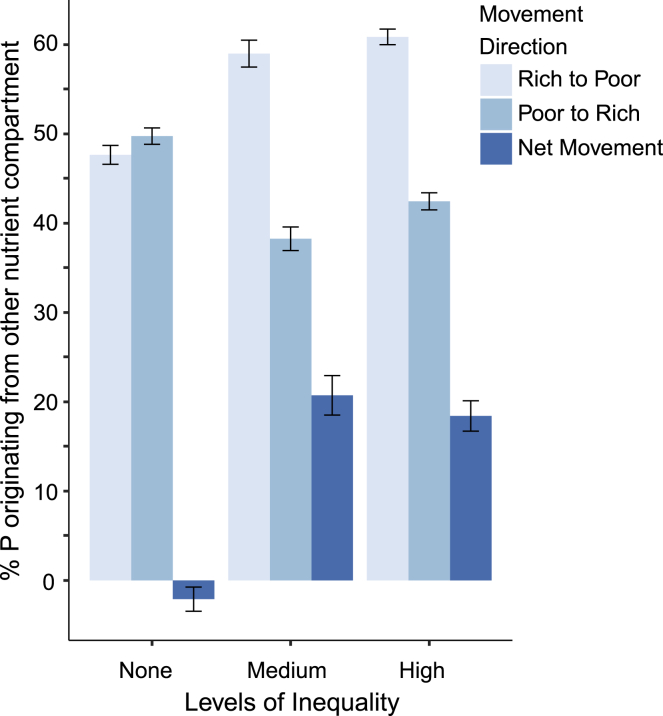
Fungi Translocate Nutrients from Resource-Rich to Resource-Poor Patches Values are expressed as the percentage of phosphorus ± SE in the fungal network that originates from the adjacent nutrient compartment. Movement of rich to poor compartments (gray) and poor to rich compartments (light blue), with net movement (dark blue) calculated as the difference between the two. Level of inequality and movement direction had a significant interaction effect (χ^2^ = 146.1, df = 2, p < 0.01), indicating an effect of inequality on net phosphorus transfer between compartments. See also Figures S2–S4.

Rather than remaining localized in a given section of the fungal network, we found that phosphorus of different origins was translocated across the fungus in both directions, from phosphorus-poor to phosphorus-rich patches, and vice versa. However, exposure to inequality was associated with a higher net movement from rich to poor patches (Figure 3). When there was no inequality, net movement between sections of the fungus was not significantly different from zero. This suggests that, as well as adjusting its level of trade with the host plant, inequality led the fungus to differentially move resources within its network, specifically from rich to poor patches.

### Tracking Particles within the Fungal Network

**Figure 4:**
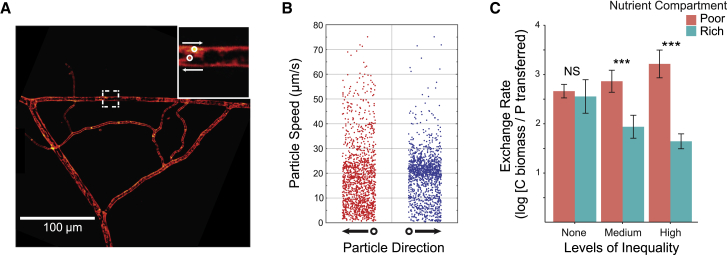
Measuring Velocity of Cellular Contents within Fungal Networks (A) Using video microscopy, we documented highly dynamic flow patterns in individual hyphae, with cellular contents reversing directions on the order of seconds (Videos S1 and S2). We then tracked individual particles (e.g., organelles; see inset) within single hyphae under control conditions with no addition of quantum dot-apatite, and extracted their velocities. Grayscale was converted to color using a color look-up (CLU) table for visual contrast. See bar for scale. (B) Speeds of particles in the living fungal network were pooled across three time points under control conditions with no addition of quantum dot-apatite, showing broad speed distributions in both directions along hyphae. Direction was assigned by the sign of the velocity vector projected onto a reference axis in the lab frame, where the negative (leftward) direction pointed toward the host root compartment, and the positive (rightward) direction pointed away (leftward n = 1,353, rightward n = 1,342). (C) Exchange rate as measured by carbon received by the fungus (mg fungal biomass) from the host root in each nutrient compartment, relative to phosphorus transferred from that nutrient compartment by the fungus to the host (nmol quantum dot-apatite). Higher bars represent increasing benefits to the fungal partner. Nutrient compartment had a significant effect on exchange rate (χ^2^ = 87.8, df = 3, p < 0.01, n = 74 plates), with fungi growing in poor patches receiving significantly greater exchange rates. There was no overall effect of inequality level (χ^2^ = 4.54, df = 2, p = 0.10). Figure shows means ± SE. See also Figure S4 and Videos S1 and S2.

A net movement of resources across the fungal network from rich to poor patches could be the result of simple diffusion processes, or active advection processes under fungal regulation. We therefore studied flow patterns of cellular contents within single hyphae under control conditions in a separate experiment using high-resolution video analysis (Figure 4A). We observed highly dynamic and heterogeneous patterns of translocation inconsistent with simple Brownian diffusion processes (Videos S1 and S2). These patterns included persistent linear transport of cellular contents and simultaneous bi-directional transport within individual hyphae (Videos S1 and S2). We also confirmed that movement patterns were highly prone to change, with oscillatory flows in individual hyphae reversing direction at intervals on the order of seconds (Videos S1 and S2).

Video S1. Close-Up of Hyphal Streaming Demonstrating Direction Reversals over Time, Related to Figures 4 and S4 Directional changes in the bulk movement of cellular content is clearly visible in this color-transformed brightfield image sequence, with no addition of quantum dot-apatite, measured under control conditions. Inversion of the streaming direction can be observed several times over the course of this 30 s video. Grey-scale was converted to color using a Color Look-Up Table (CLUT) for visual contrast.

Video S2. Streaming Direction Reversals in a Larger Network, Related to Figures 4 and S4 Bright-field imaging in region of the hyphal network experiencing directional changes in the bulk movement of cellular content, with no addition of quantum dot-apatite measured under control conditions. On the right edge of the triangle, cytoplasmic movement is observed downwards, changing direction around the 10 s mark, and changing back around 20 s. Grey-scale was converted to color using a Color Look-Up Table (CLUT) for visual contrast.

To further assess the contributions of advective and diffusive transport, we quantified these transport statistics using a particle-tracking approach based on traceable cellular contents (e.g., organelles) [27] (Figures 4A and 4B). We observed that distributions of particle speeds (v) were broad (18.8 ± 11.8 μm⋅s^−1^, mean ± SD) (Figure 4B) and complex in shape (Figure S4A), reflecting the dynamic and diverse flow patterns noted above. The speeds of the fastest-moving particles exceeded 50 μm⋅s^−1^ (Figure 4B), roughly 100 times faster than expected under Brownian diffusion, again indicating the dominance of strong advective transport (Figure S4B). Our observations of active translocation processes are in line with previous work showing that arbuscular mycorrhizal hyphae employ cytoplasmic streaming as well as a full array of cytoskeletal proteins, such as “track-forming” microtubular arrays [28], to translocate resources bidirectionally [29].

By actively regulating the movement of cellular contents [30], the fungus may be able to respond to local conditions across its network. If the fungus receives a greater carbon payoff from the host by trading in areas where the demand for phosphorus is higher, this could explain the active translocation of phosphorus to resource-poor areas. To test this hypothesis, we calculated the “exchange rate” the fungus receives in rich and poor compartments, namely carbon gained per unit of phosphorus transferred. Whereas quantum-dot-tagged phosphorus allowed us to quantify fungal transfer from rich and poor patches, measuring carbon gains from the host using quantum-dot-tagged carbon is a future goal. This remains methodologically challenging, because organisms will consume carbon at a faster rate than phosphorus, potentially exposing heavy-metal cores [31]. Alternative approaches for quantifying exchange rates, such as ^13^C labels, are useful for studying short-term carbon flow patterns [32], but a ^13^C signal will become too diluted over the timescale (60 days) of our experiments. Instead, fungal biomass is considered the best proxy for measuring long-term host allocation patterns to networks because arbuscular mycorrhizal fungi are obligate biotrophs [33]. Obligate biotrophy means the fungus cannot grow in the absence of a root—all carbon gained by the fungus to form and maintain networks is derived from the host. We therefore determined trade gains for the fungus across the network, asking how much fungal biomass is gained from the host root relative to phosphorus transferred from each nutrient patch.

We found that fungal sections growing in the resource-poor patches showed consistently higher trade gains, with increased fungal biomass per unit phosphorus transferred compared to those in the resource-rich patches (Figure 4C). These data are consistent with the idea that as phosphorus availability decreases, its net value can increase [8, 34]. As a result, the fungus may be favored to move phosphorus away from rich patches, where it would fetch a low price, to poor patches, where it potentially fetches a higher price. In the absence of inequality, we found no difference in exchange rate (50:50 treatment) (Figure 4C).

Although variation in trade gains provides a potential adaptive explanation for the translocation of the phosphorus to the poor side, more precise data on host carbon allocation patterns are needed, including how the fungus itself redistributes carbon within the network. For example, once developed, a quantum-dot-tagged carbon would allow us to spatially monitor where and when hosts allocate resources to the fungus, and to confirm variation in local exchange rates across networks exposed to inequality.

An open question is to determine how fungi integrate resource cues across their networks. Our results are in line with recent work suggesting that filamentous fungi are capable of coordinating hyphal behavior over their complex physical architectures [35]. Although the underlying molecular mechanisms remain unknown, there is mounting evidence that slime molds and networks of some fungal species use oscillatory rhythms to coordinate behavior of different hyphal types in heterogeneous environments [35, 36, 37, 38], allowing them to propagate signals and transmit efficient distributions of nutrients in the absence of a centralized control system [37].

More broadly, our results suggest that even microenvironments of resource heterogeneity can alter the way symbiotic organisms exchange resources. We found that exposure to spatial inequality can lead fungi to increase the total amount of phosphorus they trade, and also to redistribute phosphorus within their networks, from rich to poor patches. Previous research has shown how organisms, such as ant colonies [39] and modular plants [40], use networks to redistribute resources in response to their own metabolic requirements. Our work shows that symbiotic fungi likewise use networks to move resources, but also raises the possibility that this translocation reflects a mechanism to increase returns on trade from hosts. Finally, although we have validated the precision of our phosphorus-tracking methodology in a whole-plant system (Figures S2A and S2B), future work can now scale up to *in situ* whole-plant communities, where trading strategies can be studied under increasingly realistic conditions, such as when there are multiple fungal symbionts per host and the fungal network simultaneously connects multiple plants. These tests are key to understanding how organisms, outside of humans, cope with variation in local market conditions.

## STAR★Methods

### Key Resources Table

**Table:** 

REAGENT or RESOURCE	SOURCE	IDENTIFIER
**Chemicals, Peptides, and Recombinant Proteins**		

Carboxyl terminated quantum-dots 488 nm	Crystalplex	N/A
Carboxyl terminated quantum-dots 666 nm	Crystalplex	N/A
Phytagel™	SIGMA-ALDRICH	Cat#P8169

**Critical Commercial Assays**		

DNeasy Plant Maxi Kit	QIAGEN	Cat#68163
iTaq Universal Probes Supermix	BIO-RAD	Cat#1725134

**Deposited Data**		

Dryad data repository	Dryad data repository	Dryad doi: https://doi.org/10.5061/dryad.n343sh3

**Experimental Models: Organisms/Strains**		

Ri T-DNA *Daucus carota* transformed root cultures	Prof. Jan Jansa Institute of Microbiology Czech Academy of Sciences Prague, Czech Republic	N/A
*Rhizophagus irregularis* strain A5	Prof. dr. Ian Sanders, Department of Ecology and Evolution, Université de Lausanne, Switzerland	N/A
*Medicago truncatula* Gaertn	Prof. B. Hause, Leibniz Institute of Plant Biochemistry, Halle, Germany	N/A

**Oligonucleotides**		

Forward primer internal standard: CGAACCTGGACTGTTATGATG	[17]	N/A
Reverse primer internal standard: AATAAACAATCCCCTGTATTTCAC	[17]	N/A
TaqMan probe internal standard: CACCAGGCACCAACAACGACCATT	[17]	N/A
Forward primer R. irregularis: TTTTAGCGATAGCGTAACAGC	[17]	N/A
Reverse primer R. irregularis: TACATCTAGGACAGGGTTTCG	[17]	N/A
TaqMan probe R. irregularis AAACTGCCACTCCCTCCATATCCAA	[17]	N/A

**Software and Algorithms**		

SimFCS version 4 software	Laboratory for Fluorescence Dynamics, University of California, Irvine, USA	N/A
Fiji	[41]	N/A
R 3.4.4	R core	N/A
MATLAB R2016a	Mathworks	N/A
Bio-Rad CFX Manager Software	Bio-Rad	N/A
TrackMate	[27]	N/A

**Other**		

Petri dishes- 90mm, 3 compartments, vents	VWR	Cat#710-3518
Petri dishes- 90mm, 2 compartments, vents	VWR	Cat#710-3509
Whatman Membrane filter, cellulose-nitrate, 45 μm, 47 mm	SIGMA-ALDRICH	Cat#10401112
Cell imaging plates, 96-well glass bottomed	Eppendorf	Cat#30741030
Thermo Savant FastPrep Fp120 Cell homogenizer	Thermo	N/A
Olympus Confocal Laser scanning microscope, FluoView FV1000	Olympus	N/A
Olympus UPLSAPO 60 WX objective, water immersed	Olympus	N/A
Quadro dichroic mirror: 405/488/560/635	N/A	N/A
Emission filter: 455-500 nm	N/A	N/A
Emission filter: 610-710 nm	N/A	N/A
Pulsed laser, 20MHz, 405 nm	PicoQuant	N/A
BioTek Synergy MX plate reader	BioTek	N/A
Bio-Rad CFX96 Lightcycler	BIO-RAD	N/A
Nikon TE2000-U inverted optical microscope	Nikon	N/A
Nikon 40x phase contrast objective, S Plan Fluor ELWD, 40, Ph2	Nikon	N/A
PCO.edge sCMOS camera	PCO.	N/A

### Contact for Reagent and Resource Sharing

Further information and requests for resources and reagents should be directed to and will be fulfilled by the Lead Contact, E. Toby Kiers (toby.kiers@vu.nl)

### Experimental Model and Subject Details

#### *In-vitro* Root Organ Culture experiments

For all *in-vitro* experiments, we utilized Ri T-DNA Carrot (*Daucus carota*) transformed root cultures grown *in-vitro* on Modified Strullu-Romand (MSR) media [17, 21, 22]. Each experiment was initiated with a 2cm long root segment of *D. carota* cultures growing for three weeks. We transferred root segments to Petri dishes divided into either two or three compartments depending on the experiment. We filled each compartment with an adjusted MSR medium, depending on the particular experiment, consistently keeping the pH at 6. We then inoculated each root with a 1.5 cm x 1.5 cm inoculation plug of a single *R. irregularis* strain A5, containing hyphae and roughly 400 spores per plug [22]. We kept all plates in a climate-controlled chamber, in the dark at 25°C.

#### Whole plant experiments

For all whole plant experiments, we used *Medicago truncatula* Gaertn. (courtesy of Prof. B. Hause, Leibniz Institute of Plant Biochemistry, Halle, Germany) as a host and germinated seeds as described [42, 43]. After germination, we transferred the seeds to pots containing 160 g autoclaved seedling soil, and cultivated them in a climate-controlled room (16 hours in the light at 22°C interspersed by 8 hours in the dark at 17°C; 75% humidity). After 21 days of cultivation, we transferred seedlings to split pots so that the root systems could be split equally into different nutrient treatments on the same plant.

Each pot was fitted with a sealed divider to create two equally sized compartments. Each pot contained one plant, with its roots equally divided between the two compartments. Each compartment contained 160 g of autoclaved quartz sand. To each root half, we added 5 mL of Hoagland’s solution and 1 mL of *R. irregularis* inoculum (1000 spores/mL). The control plants received 5 mL of Hoagland’s solution and 1 mL of water, but no fungi. We pipetted this liquid across the roots in both compartments and then covered the roots with a layer of autoclaved and sterile low-density polyethylene beads. We maintained a 50% water saturation by watering twice a week with demi-water based on pot weight.

### Method Details

#### Conjugating hydroxyapatite with quantum dots

We tagged hydroxyapatite, a form of rock phosphate [44, 45], with fluorescent nanoparticles. Specifically, we used two spectrally different carboxyl terminated quantum-dots (QDs) with 488 nm cyan and 666 nm red emission (Crystalplex, Pittsburg PA, USA). These quantum dots were selected because of their small size (< 5.5 nm diameter), and composition-tuned CdSeS cores that are wrapped in ZnS shells. Composition tuning means that the emission colors are based on core compositions rather than QD size. As a result, our QDs were equivalent in size and mass, independent of emission color. We synthesized the coated quantum-dot nanoparticles (QD-apatite) using a modified simulated body fluid (MSBF) method [46, 47]. The carboxyl terminals (i.e., oleic acid based protective coatings) of the QDs served as an anionic binding site to coat the QDs in apatite via the MSBF. Specifically, each reaction mixture consisted of 499.5 nmol QD in 1L of nanopure water reacted against a commonly used MSBF solution composed of 11.992 g NaCl, 1.966 g NaHCO_3_, 0.447 g KCl, 0.458 g MgCl_2_∙_6_H_2_O, 0.261 g K_2_HPO_4_, 0.416 g CaCl_2_, and 0.107 g Na_2_SO_4_.

We conducted the reactions in two phases. We performed the initial reaction in the dark at 37°C for 24 hours under static conditions. We then cooled the reaction to room temperature and mixed for 24 hours on a gentle shaker to obtain an initial set of QD-apatite crystals (∼8 nm diameter). After this initial reaction, the mixture was once again heated to 37°C for 120 hours under static conditions to obtain final QD-apatite crystals of ∼200 nm diameter, which were composed of smaller 8 nm crystals (Figure S1A). This bigger composite aggregate, comprising smaller crystals, closely mimics apatite crystals found in nature [48]. To remove any unbound MSBF, we replaced the supernatant of each reaction mixture with sterile water three times. We then shook the reaction mixtures by hand and allowed them to re-precipitate between each wash. After washing, we wrapped reaction vials in foil, and stored them at 4°C in the dark. Transmission electron microscopy (TEM) was performed in collaboration with the Electron Microscopy Center Amsterdam, core facility Cellular Imaging (Figure S1B). We determined the surface structure of the crystalized QD-apatite using X-ray photoelectron spectroscopy (XPS), performed by Evan Analytical Group EAG (East Windsor, NJ, USA). Photoelectrons were generated within the X-ray penetration depth of 5-10nm over a 2.0 mm x 0.8 mm surface of dried QD-apatite using a PHI 5701LSci fitted with a Monochromated Alk_α_ 1486.6eV xray source. Detection limits were approximately 0.05 to 1.0 atomic %. Using this analysis, we determined that each nmol of QD-apatite contained ∼700 nmols of P (nmol P:QD = 708:1, Figure S1C).

#### Experimental design *in-vitro* Root Organ Culture

Carrot was chosen as a host for our *in-vitro* experiments because it shows a high mycorrhizal dependency, and is a model organism for studying AM mycorrhizal interactions [49]. Root organ cultures provide similar C sources (hexoses and lipids) to fungi as roots in nature: substrate transfer from host to AM fungi in both whole plant systems and monoxenic root-organ cultures depend on the host root converting carbon backbones to hexoses and lipids [33, 50]. AM fungi are obligate biotrophs, as well as fatty acid auxotrophs [50, 51].

For our inequality experiment, we grew root organ cultures in Petri-dishes containing three separate compartments (Figure 1A). Each compartment contained 14.6 mL of MSR. The compartment with the root culture contained standard MSR, while the two hyphal compartments contained MSR lacking phosphorus resources. Three weeks after fungal inoculation, hyphae from the colonized root successfully crossed the plastic lip into the two separate hyphal compartments. Because of a plastic barrier between the two hyphal compartments, the hyphae remained in the compartments containing their individual resource patches [52]. However, the fungal network itself was continuous and connected via the root compartment (Figure 1A).

We added a total of 0.18 nmol QD-apatite to the hyphal compartments. The QD-apatite concentration was chosen such that the media would not be depleted by the end of the 60-day experiment. Under these conditions, the host maintains a sustained demand on the fungus for phosphorus needs, while continuing to grow. These conditions ensure that the host is neither overly saturated with nutrients (toxic range) nor completely deficient [17, 22, 53].

Following hyphal crossover, we injected a total of the 0.18 nmol of QD-apatite into the two hyphal compartments in three ratios: the no-inequality (50:50) treatment received 0.09 nmol cyan QD-apatite in one compartment and 0.09 nmol red QD-apatite in the second hyphal compartment; the medium inequality (70:30) treatment received 0.126 nmol cyan QD-apatite in one hyphal compartment and 0.054 nmol red QD-apatite in the other; and the high (90:10) inequality treatment received 0.162 nmol cyan QD-apatite in one hyphal compartment and 0.018 nmol red QD-apatite in the other. Each of the inequality treatments was replicated 30 times.

In a separate experiment, we tested the relationship between nutrient availability and fungal P transfer to host using a two-compartment setup. We grew colonized roots in a three-compartment Petri plate as above, but one compartment was left media free. This allowed us to restrict hyphal growth to only one compartment containing a single phosphorus level. As above, the hyphal compartments were injected with QD-apatite solutions equaling the individual treatment compartments as above (10 = 0.018 nmol QD-apatite; 30 = 0.054 nmol QD-apatite; 50 = 0.090 nmol QD-apatite; 70 = 0.126 nmol QD-apatite; 90 = 0.162 nmol QD-apatite). Each of the five nutrient concentration treatments was replicated 12-17 times. As expected, we found that phosphorus transfer to the host increased linearly with increases in P concentrations (Figure S3D), confirming that the ranges of P used are appropriate to study trade dynamics.

To confirm that AM fungi do not grow in the absence of a host (e.g., gaining carbon directly from the media), and that P concentrations in the media do not affect this process, we ran an experiment in which fungal plugs were cultured on MSR either with, or without, phosphorus. Specifically, hyphal plugs (1.5 cm x1.5 cm) containing networks of living hyphae of *R. irregularis* were cut and placed on solid media containing 1% Phytagel. We added either 500 ul water or apatite solution to the media before adding the hyphae plug. We used a total of 15 replicates for each condition (30 plates total). In 29 of 30 plates started from fungal plugs, we detected no evidence of fungal growth in either nutrient treatment confirming their obligate dependency on hosts for network formation. In one case, we found that a single hypha showed evidence of elongation, but this remained < 1cm over the 5-week period.

We ran a similar experiment with individual spores to determine their growth patterns in the absence of a host, and to ask whether growth differed when apatite was added to the media. We isolated 214 spores of *R. irregularis* grown from *D. carota* ROC and added these to 28 replicate plates in 126 spore groups (∼4.5 groups per plate, each containing an average of 1.7 spores). The 28 replicate plates were divided in two treatments: apatite solution or water. We imaged spore growth every week for over 5 weeks to determine whether spores could grow and form networks in the absence of a host root. We found that 18.3% of spore groups showed evidence of germination (in water: 19.6%, in apatite 17.1%). Germination of spores was generally limited to hyphal growth of < 2cm, with a maximum growth of 2.2 cm. This indicates that in the absence of host, spores can germinate from carbon stored in the spores, and form initial exploratory hypha but cannot build a network.

#### Quantum Dot diffusion, color, and unconjugated control experiments

To determine if there was any non-biological movement of the QD-apatite across the plastic barriers, we conducted two diffusion control treatments. First, we used a non-inoculated *in-vitro* root in which the host roots were constrained to one compartment containing MSR-media. The second compartment contained MSR-media, but neither roots nor hyphae. We then added 0.09 nmol QD-apatite to the non-colonized hyphal compartment. This set-up was replicated 8 times, and harvested 60 days after injection. We found no signal of QD-apatite (i.e., values lower than the detection limit; < 0.000001 nmol QD mg^-1^ plant tissue) in the root tissue, confirming there was no non-biological movement of QD-apatite across the plastic barrier.

In a second diffusion control, we tested for QD-apatite movement across non-connected, severed hyphal tissue. We inoculated the *in-vitro* root as above, but did allow the hyphae to cross into the hyphal-only compartment containing MSR-media. Thirty days after hyphal cross-over, we manually cut the hyphae and immediately injected 0.09 nmol QD-apatite into the hyphal compartment. This was replicated 9 times, and harvested 60 days after injection. We found no uptake of QD-apatite (i.e., values lower than the detection limit; < 0.000001 nmol QD mg^-1^ plant tissue) in the root tissue, confirming the hyphae need to be connected and living for translocation of QD-apatite into the host root.

To determine if there were any differences in uptake and transfer affinity between the two colors of QD-apatite, we conducted a color control in which two colors of QD-apatite were added to the same compartment. We then tested for a color bias. We inoculated *R. irregularis* on an *in-vitro* root grown in a two-compartment Petri dish. Once the hyphae had crossed into the hyphal compartment (∼30 days), we injected equal amounts (0.2 nmol) of 488 nm cyan and 666 nm red QD-apatite in the same hyphal compartment (0.4 nmol QD-apatite in total). Before injection, only plates that match our exact criteria of uniformity were retained. Criteria included (i) no root cross-over, (ii) similar densities of hyphae on fungus-only side, (iii) similar distributions of hyphae across space, and (iv) no contaminations from other microorganisms. In total we had 5 replicates, which were harvested 60 days after injection. While our statistical power to detect differences was small because of high variation at the replicate level, we found no indication of differences in QD-apatite transfer based on color (Figure S2A). This color control complimented three other independent tests to confirm that color did not influence uptake of QD-apatite (see Figures S2B and S2C and Table S1).

To determine if there was an affinity for unbound (i.e., unconjugated) QDs that lacked apatite, we inoculated an *in-vitro* root grown in a two-compartment Petri dish. Once the hyphae had crossed into the hyphal compartment (∼30 days), we injected 0.2 nmol of 488 nm cyan unconjugated carboxyl terminated QDs into the hyphal compartment. This was replicated 5 times and harvested 60 days after injection. We found no uptake (i.e., values lower than the detection limit; < 0.000001 nmol QD mg^-1^ plant tissue) of carboxyl terminated QDs lacking apatite.

Finally, to test for artifacts associated with the uptake and movement of bare metal QD cores (i.e., ‘naked quantum dots’ lacking their protective carboxyl-terminated oleic acid surface polymers), we inoculated an *in-vitro* root grown in a two-compartment Petri dish. After 76 days of growth, we injected 0.2 nmol of bare metal (CdSeS/ZnS) QDs into the hyphal compartment. This was replicated 6 times and harvested 32 days after injection. We found no uptake (i.e., values lower than the detection limit; < 0.000001 nmol QD mg^-1^ plant tissue) of bare metal naked-QDs.

While more work is needed to characterize the specific uptake pathways for QD-apatite, previous studies have shown that QD-tagged nutrients (e.g., QD-tagged glutathione) can be actively taken up by fungi using specific membrane transporters (e.g., ADP1-encoded transporters in yeast cells) [54]. In knockout strains in which the specific ADP1 glutathione permease was removed, cellular uptake of QD-labeled glutathione decreased by ∼95% [54]. For apatite particles, uptake by fungi is thought to involve high affinity Pi transport pathways [55, 56, 57, 58]. AM fungi have also been shown to take up QD-tagged organic nutrients - as large as 30 nm in diameter [59, 60]. For even larger particles, active endocytic pathways are potentially exploited. For example, clathrin-coated vesicles (i.e., receptor mediated endocytosis) can accept particles up to 120 nm in diameter [61, 62, 63] and other, non-selective forms of endocytosis (i.e., fluid phase endocytosis) can take up nanoparticles and nutrients up to 500 nm in diameter [61, 63, 64, 65, 66].

#### Harvest of *in-vitro* root organ cultures

In all inequality experiments, we harvested cultures 60 days after QD-apatite injection, flash froze and stored them at −80°C. We discarded replicates exhibiting any signs of bacterial or fungal contamination. We harvested roots from each compartment using forceps, rinsed roots to ensure no material remained on the outside, and then oven dried them at 60°C for 72 h. We then measured dried root biomass (mg) on an analytical balance (Figure S3A). We homogenized the dry roots and removed subsamples for DNA extraction and QD-apatite fluorescence analysis. We visually determined the root colonization percentage using a subset of randomly-selected 1 cm root samples [67]. Colonization by AM fungi was assessed based on the presence of arbuscules and characteristic intraradical hyphae with irregular walls, angular branching and lack of septa. We found no significant differences in intraradical colonization across the treatments (Figure S3B).

We collected and rinsed the extraradical hyphal network from each compartment by first dissolving the phytagel-based media in 10 mM sodium citrate at 65°C, and then collecting the AM fungal tissue on a 0.45 μm filter membrane under vacuum [22]. After filtration, we freeze-dried the extracted AM hyphae for 24 h before DNA and QD-apatite analyses. We extracted fungal DNA following [53]. We used a probe and primer pair specific to the lesser subunit of mitochondrial DNA of *R. irregularis*, with a 2x concentrated iTaq Universal Probes Supermix (BioRAD) to prepare the PCR mix. For each sample, we added 16 μL of the PCR mix and 4 μL of the isolated DNA in a well of a 96 wells plate. Additionally, if the qPCR relative fluorescence was not logarithmic, DNA samples were diluted until 100% PCR efficiency was recovered. We performed all qPCR analyses using a CFX Connect Real-Time PCR Detection System from Bio-Rad. We then transformed Cq values into raw copy numbers and used the DNA isolation efficiency to normalize the raw *R. irregularis* copy numbers. Probe design, qPCR calibration, detection limits, and plasmid preparation are as described in [53]. To convert copy numbers to biomass, we created a calibration curve (R^2^ = 0.70; N = 26) of AM hyphal biomass against their corresponding copy numbers determined by qPCR. This approach was re-validated by recent work testing the abundance and marker relationship when looking within a single AM fungal isolate [68], where it was demonstrated that mtDNA markers generate well-correlated abundance data and can be used successfully to quantify particular isolates. In addition, we avoided any issues regarding variation in the concentration of mitochondria by harvesting the entire hyphal network for our measurements, rather than a fraction (Figure S3C).

#### Fluorescence analysis: microplate reader

We analyzed subsamples of hyphae and roots containing QD-apatite for fluorescence intensity. Using samples in which surface nutrients were removed, we manually homogenized roots and separated them into 5 subsamples for microplate readings. To prepare samples, we added borate buffer (10 mM at pH 7.4) to each sample to maintain a ratio of 1 mg dry root per 150 μL buffer, and placed 1mg of each subsample into a 96-well glass bottomed plate (Eppendorf AG, Hamburg, Germany). We obtained spectra from each sample-well in the wavelength range of λ = 450-800 nm with 2 nm intervals using a Bio-Tek Synergy MX plate reader at 325 nm excitation (BioTek Instruments, Bad Friedrichshall, Germany).

To quantify the abundance of QD-apatite within root tissue, we first confirmed that the QD-apatite was inside the root tissue using confocal microscopy (Figure S1D) for example of visualization), and then conducted emission fingerprinting. This technique allows multiple fluorescence spectra (including plant autofluorescence) to be unmixed from a single spectral scan of a sample, which may be composed of similar or even widely overlapping emission spectra [69]. Our script takes into account the high plant autofluorescence in our system, and allows us to analyze low levels of QD-apatite (∼0.000001 nmol QD mg^-1^ plant tissue) that would be difficult to detect using traditional filter and channel-based techniques.

We next created reference spectra for cyan QD-apatite, red QD-apatite, root autofluorescence, and borate buffer backgrounds (Figure S1G). Before spectral unmixing, we subtracted absolute backgrounds from each sample spectra [70, 71]. These reference spectra were then used to unmix cyan QD-apatite, red QD-apatite and plant autofluorescence components from the spectral scans of each sample spectrum, using linear regression (Figure S1G) [72, 73, 74]. After spectral unmixing, the resulting curves were smoothed to further reduce noise. We then summed the photon counts of each QD color across each unmixed curve, and converted these counts to specific uptake (nmol QD-apatite mg^-1^ plant tissue), using a total photon calibration curve composed of eight QD concentrations.

#### Fluorescence analysis: Confocal analyses

To assess uptake and storage of QD-apatite by AM fungi, we used Raster Image Correlation Spectroscopy (RICS) [60, 75, 76, 77]. This technique allows the quantification and visualization of QD-apatite within cytosol and vacuoles in AM hyphae. We conducted all confocal analyses using an Olympus FluoView FV1000 confocal microscope with a water immersed 60x UPLSAPO objective. Excitation was conducted using a 20 MHz pulsed 405 nm laser (Picoquant, Berlin, Germany). We collected fluorescence by internal photo multiplier tubes using a 405/488/560/635 quadro dichroic mirror in combination cyan (455-500 nm) & red (610-710 nm) emission filters. We analyzed the collected images using SimFCS version 4 software (Laboratory for Fluorescence Dynamics, University of California, Irvine, USA).

We scanned images (4.096 μm x 4.096 μm) at 50x zoom with individual pixel sizes of 16 nm. An optimal pixel dwell time of 20 μs pixel^-1^ was used at a raster line speed of 1.248 ms. Before calculating image functions for quantification, we conducted moving average subtractions (4 frames) to remove background and non-moving hyphal autofluorescence [78]. The beam waist size corresponds to 0.250 μm as measured by fluorescence correlation spectroscopy (FCS).

On the harvested network, we performed RICS on eight randomly selected extracted AM hypha from each compartment in each replicate. To confirm the QD-apatite was within the AM fungal hypha, we scanned with a focal depth of 1.5um (Figure S1F). We collected 40 images (256 × 256 pixels) per hyphal location (vacuole or cytosol). The RICS autocorrelation function [60] was calculated to extract the number of particles in the excitation volume (biovolume hypha). We estimated QD-apatite (QD particles absorbed μm^-3^ hypha) retention from the number of QD particles present per biovolume tissue visible per confocal scan. We calculated retention (nmol QD-apatite per total hyphae) as the product of hyphal biomass per compartment and specific uptake by extraradical AM hyphae. For extraradical AM hyphae, we assumed a fresh hyphal density of 1.1 g cm^-3^ and a water weight content of 60% [79].

To quantify relative storage across the fungal network, we measured the percentage of vacuole area per total hyphal area. Using Fiji ImageJ and a FluoView FV1000 confocal microscope, we collected four white light images from each replicate [41]. In each image, we measured total hyphal area and then measured total area vacuole by adjusting the color threshold to outline and select the vacuoles within each frame. Percent vacuole was calculated by dividing the total area vacuole by the total area hyphae, and multiplying by 100.

#### Whole plant experiments

We further validated our QD-apatite fluorescence method using *M. truncatula* plants. Whole plant validation of our method is important because of the lack of photosynthetic tissues in *in-vitro* cultures [21, 80]. This allows us to quantify QD-apatite movement from a sand substrate into the leaves and shoots of plants and builds on past work that has begun to characterize uptake pathways for QD-tagged nutrients in plants [63, 65, 81, 82, 83, 84, 85]. For plant roots, receptor-mediated endocytosis is likely the most common endocytosis mechanism [82]. Under this scenario, the QD-labeled nutrients are taken up and transferred to the vascular tissue of the root system, and then are transferred into shoots, mesophyll cells and even chloroplasts [59] following natural nutrient pathways. Importantly, the tagged nutrients are not stored where heavy metals typically accumulate in plant tissue [59, 86, 87], evidence that they are non-toxic.

We therefore asked whether: (1) QD-apatite was taken up by whole plants and whether a fungal network facilitated this process, (2) tagged nutrients accumulated in host shoots as expected, (3) if there was a color bias of QD-apatite in a whole plant system, (4) if there were toxic side effects. We employed a split root approach in which root halves grew in physically separated compartments containing a sand substrate. Plants were either inoculated with AM fungi or left as un-inoculated controls. Root halves were then injected with either cyan QD-apatite (488 nm), and red QD-apatite (666 nm) in two experiments, either in 50:50 (Figure S2B) or 90:10 ratios (Figure S2C). Under these conditions, both the roots and fungal network are grown together in a single sand substrate.

We grew the plants for 22 days before injecting root halves with either 488 nm cyan emission QD-apatite or 666 nm red emission QD-apatite. Root halves received 5.09 nmol QD-apatite per root compartment resulting in a 50:50 ratio of the two colors. Plants were grown with 25 mL of Hoagland solution per pot [88] with P content reduced to 50% of the standard Hoagland’s solution [43, 89]. We confirmed that in our non-mycorrhizal treatment, our whole plants showed no colonization by fungi. In a second experiment, we again grew roots with split root systems and added a total of 10.2 nmol QD-apatite to the pot (same total amount as 50:50 experiment). However, this time each root half received QD-apatite in a ratio of 90:10. This allowed us to reverse the association between cyan and red QD apatite in rich (90) and poor (10) treatments.

We also tested for QD-toxicity in whole plants in a second experiment where we compared QD-apatite to apatite lacking a QD tag (Figure S2D). Toxicity is thought to be prevented because the protective polymers on the QDs remain intact— successfully “hiding” the heavy metal cores of the QDs. In the absence of protective surface QD polymers (or if the protective polymers become compromised), toxicity will start to appear in living tissue [84, 85]. Specifically, we were interested in whether exposure to QD-apatite directly affected plant growth patterns. We therefore compared *M. truncatula* mycorrhizal plants grown with QD-apatite to apatite lacking QD-cores (i.e., not conjugated to any QDs). To synthesize apatite lacking QD cores, we used the same reaction process as described above, except using 999 nM citric acid as a binding surface instead of carboxyl terminated QDs. We performed all chemical reactions in the dark.

We grew 15 replicate plants as above (but lacking root dividers) and injected them with either 10.2 nmol QD-apatite or 10.2 nmol apatite lacking QDs. We grew plants for 49 days before harvesting. At harvest, we cut plant shoots, separated them from roots at soil level in each pot. We removed roots from each compartment using a 0.5 mm sieve, washing them 3 times with demineralized water. We dried roots and shoots at 60°C in a drying oven for 72 hours. We measured dry root and shoot biomass (mg) on a microbalance.

#### Whole plant florescence analysis

To quantify the abundance of QD-apatite within shoots and roots of *M. truncatula*, we again conducted emission fingerprinting. Because of the high amount of plant autofluorescence in our system, we created reference spectra for cyan QD-apatite, red QD-apatite, root and shoot autofluorescence of *M. truncatula*, and borate buffer backgrounds, as above (Figure S1H). Before spectral unmixing, we subtracted absolute backgrounds from each sample. These reference spectra were then used to unmix cyan QD-apatite, red QD-apatite and plant autofluorescence from the spectral scans of each sample. Five subsamples of each replicate were analyzed and averaged per sample. To minimize edge effects, the outer most cells of each plate were not used. As described above, spectral scans were obtained in a wavelength range of 450–800 nm with 2 nm intervals using a Bio-Tek plate reader at 325 nm excitation.

We regressed the resulting calibration ratios against the known QD-apatite concentrations to convert photon counts (A.U. mg^-1^ dry plant tissue) to specific uptake (nmol QD-apatite mg^-1^ dry plant tissue) and then calculated total QD uptake per sample (nmol QD-apatite per total dry root/shoot) as the product of specific uptake of plant tissue and absolute biomass per sample.

We confirmed that QD-apatite was taken up by whole plants, and translocated into the leaves and shoots, as expected under natural conditions. We confirmed that QD-apatite fluorescence was retained in the shoots. We also confirmed that inoculation with AM fungi facilitated the uptake of QD-apatite in plants compared with non-mycorrhizal controls (t = 3.11, df = 37.3, p = 0.003), as found previously with naturally occurring apatite [44, 90]. We pooled data from mycorrhizal and non-mycorrhizal treatments and confirmed there was no color bias in the uptake of QD-apatite in the 50:50 split root treatment (Figure S2B).

In our color-switching whole-plant experiment, we found that mycorrhizal plants contained more QDs originating from the high resource compartment (90 compartment) than from the low resource compartment (10 compartment), as expected (Figure S2C). There was no indication for an effect of source color on QD-uptake by plants, further confirming that colors are taken up and transferred to the host plant equally well (Figure S2C).

In our whole-plant toxicity controls, we also found that there were no significant differences in root or shoot growth when comparing the QD-apatite and natural apatite that was not conjugated to any QDs (Figure S2D).

#### Measuring advection within hypha using particle track

We ran a separate experiment to determine average velocities of cellular contents moving inside a living hyphal network (Figure 4). As above, we grew *in-vitro* Ri T-DNA *D. carota* transformed root cultures on 14.6 mL standard MSR media to measure transport by advection. Roots were confined to one compartment, and hyphae of the fungus *R. irregularis* A5 crossed into a hyphal-only compartment lacking QD-apatite. We grew plates for 8 weeks until fungal networks were established. For each plate, we selected a minimum of six fields of view (FOV) with clear presence of fungal networks.

We placed each plate on a custom support, and then imaged hyphae using a Nikon TE2000-U inverted optical microscope equipped with an air condenser (Nikon LWD, numerical aperture (NA) 0.52) and a 40x phase contrast objective (Nikon, S Plan Fluor ELWD, 40, Ph2, NA 0.6). For each field of view, we recorded 200 frames at 10Hz and an exposure of 30ms using a PCO.edge sCMOS camera.

We imaged the network of six replicate hypha at three time points (0, 30 and 60 minutes) under control conditions (no QD-apatite added) to obtain movies suitable for particle tracking. We processed each set of images using FIJI [41] to identify traceable cellular contents. This included image acquisition, pre-processing (e.g., noise removal with Gaussian Filter, filtering with FFT), segmentation (e.g., particle identification, threshold/binarization), particle tracking and data analysis. The processing steps were performed in all movies independently. We applied an FFT bandpass filter to remove structures larger than 40 px and smooth features smaller than 3 px. For a given movie, we obtained, and then projected, a minimal pixel value as a reference for background. We then subtracted this reference from the original stack.

To track single particles, we implemented an automated pipeline using TrackMate [27] to identify and follow cellular content. In our images, each traceable cellular content was a 10px object. Since the identified features move along the hyphae, this spatial constraint, allowed us to use a Kalman Filter as implemented on TrackMate. This filter searches for particles that move linearly in space. Particles moving perpendicular to the hyphae length were not observed. Tracks of length < 5 frames were excluded from the analysis, for reliable speed estimation, and those with end-to-end distance shorter than 10px (the size of tracked objects) were excluded to filter out immobile objects. Particle speeds were separated by direction, which was assigned by the sign of the velocity vector projected onto a reference axis in the lab frame, where the negative (leftward) direction pointed toward the host root compartment, and the positive (rightward) direction pointed away.

We pooled the three time points and found a multimodal speed distribution that could be described by a sum of three normal distributions (truncated at zero), corresponding to low-, intermediate- and high-speed fractions (Figure S4A), respectively centered at ∼5 μm·s^-1^ (low-mobility), ∼20 μm·s^-1^ (intermediate-mobility), ∼30 μm·s^-1^ (high-mobility). See legend of Figure S4A for full list of fit parameters.

#### Transport estimates for advection and diffusion

To address whether the observed nutrient distribution could arise from Brownian diffusion within the hyphal network, we considered the expected displacement for phosphorus tagged with quantum dots over the timescale of our experiments (Figure S4B). We show that the expected time for a quantum-dot particle to travel the minimum distance (*L*_min_ = 3cm) to the other fungal compartment greatly exceeds the duration of the experiment if it were transported by Brownian diffusion alone. We further illustrate how advection (which in hyphae includes both cytoplasmic streaming and molecular motor transport) dominates over diffusion on the timescale of the experiment.

To estimate how fast a quantum-dot particle can move, we calculated the diffusion constant for small spheres from the Stokes-Einstein relation,

$$D=\frac{k_{B}T}{6\pi \eta a}$$

where *a* the radius of the particle, η is the viscosity of the fluid, *T* is temperature and *k*_B_ is the Boltzmann constant. Since quantum-dot particles are carried inside a hypha, we can calculate the characteristic time required for a particle to be transported over a certain a distance.

For diffusive transport, the mean squared displacement scales linearly with time (*t*) as:

$$r^{2}=2Dt$$

For advective transport, the scaling is instead quadratic:

$$r^{2}={\left(vt\right)}^{2}$$

where *v* is the advective speed of the particle.

For our experiments, the relevant parameter values are:

η = η_cytosol_ ∼η_water_ = 10^−3^ Ns/m^2^ [91]
*a* = QD size (*a*_qd_); 4 nm ≤ *a*_qd_ ≤104 nm
*t* = Harvest time (*T*_H_) ≈60 days
*r* = Minimum length to cross to the other compartment (*L*_min_) ≈3 cm

From the measured range of quantum-dot sizes, we estimate the diffusion coefficient for phosphate conjugated with QD for the extreme cases by plugging the values above into the Stokes-Einstein relationship. We then have, for the extreme cases:

$$2\mu m^{2}s^{-1}\leq D\leq 50\mu m^{2}s^{-1}$$

from which, we can then estimate the expected diffusive transport time $\tau \approx L_{min}^{2}/2D$, which then falls in the range:

100days≲τ≲2600days

Thus, the lower bound of the time required to cross the barrier by diffusion is ∼2-fold greater than the time of our experiment (60 days), and we conclude that it is highly unlikely that the observed transport of quantum dots is due to Brownian diffusion.

To further confirm that the transport we observed was dominated by advection rather than diffusion, we compared the expected displacements in the timescale of our experiments. We used the fastest expected diffusion coefficient (*D*_max_ = 50 $\mu m^{2}s^{-1}$), paired with a highly conservative speed for advective transport (*v*_min_ = 0.1 $\mu m\;s^{-1}$),a speed ∼3-fold lower than the lowest observed particle speed (0.32 $\mu m\;s^{-1}$). We then plotted the expected displacements of diffusion versus advection (Figure S4B). This demonstrated that the upper bound for distance traveled by diffusion is dwarfed by the lower bound for the distance traveled by advection on our experimental timescale (defined by the harvest time *T*_H_).

### Quantification and Statistical Analysis

We studied the effect of inequality on overall phosphorus transfer to the host (Figure 2A) and on phosphorus retention in the hyphal network (Figures 2B and 2C). We analyzed Gamma generalized linear models with an identity link using QD uptake as response variable and plate inequality level as categorical explanatory variable (none, medium inequality, high inequality) [92]. For the phosphorus transfer to the host, we analyzed QD concentration in the plant root (nmol QD-apatite per mg root). To study hyphal phosphorus retention, we analyzed the total amount of QD-apatite (nmol) across the total hyphae and per mg hyphae. For each inequality level, we calculated the total relative contribution of both fungal compartments as a percentage of the total phosphorus transfer, respectively retention (Table S1). We further analyzed phosphorus retention in fungal hyphae at the compartment level by generating a linear mixed model of relative vacuole surface area (%), using nutrient compartment as an explanatory variable. We analyzed a quadratic model, and to control for non-independence of compartments connected to each other on the same experimental plate, we included plate as a random intercept term [93].

To study distribution patterns, for all inequality levels, we established the percentage of QD-apatite taken up by the hyphae in both fungal compartments (rich versus poor). We also calculated the percentage of phosphorus in the hyphae that originated from the other fungal compartment. Specifically, we measured the percentage of phosphorus that originates from the other compartment, relative to the total amount of phosphorus in the hyphae. This allowed us to calculate the net movement between the rich and poor compartments (Figure 3). We analyzed the effect of inequality on the direction of phosphorus exchange by evaluating a generalized linear mixed model of QDs originating from the other compartment with direction of transfer, inequality level and their interaction as explanatory variables, and a random intercept for each plate to account for statistical non-independence among compartments connected to each other on the same plate. A significant interaction would indicate an effect of inequality on the net transfer of phosphorus among compartments.

To determine exchange rates per inequality level and resource compartment, we calculated the natural logarithm of the ratio of fungal hyphal biomass in mg (i.e., carbon received) divided by total amount of QDs transferred (i.e., phosphorous transferred) to the root (Figure 4C). We analyzed a generalized linear mixed-effects model of this exchange rate with a Gamma error distribution and an identity link function. We used inequality level and nutrient (rich, poor) nested within inequality level as explanatory variables, fitting a random intercept for each plate.

### Data and Software Availability

All statistical analyses were performed in R 3.4.4. We detail our full analyses, including all diagnostics plots, in an R-Markdown report. Our scripts and analyses are publicly available through a GitHub repository (https://github.com/gijsbertwerner/Mycorrhizal_inequality) and in a Dryad data repository (doi:https://doi.org/10.5061/dryad.n343sh3).

## Supplemental Information

Document S1. Figures S1–S4 and Table S1

Document S2. Article plus Supplemental Information
