# Supplementary material for: Mycorrhizal Fungi Respond to Resource Inequality by Moving Phosphorus from Rich to Poor Patches across Networks
Source: Curr Biol. 2019 Jun 17;29(12):2043–2050.e8. doi: 10.1016/j.cub.2019.04.061 (PMC6584331; doi:10.1016/j.cub.2019.04.061)
Supplement: Document S1. Figures S1–S4 and Table S1 [file mmc1.pdf]

Current Biology, Volume *29*

Supplemental Information

Mycorrhizal Fungi Respond to Resource Inequality by Moving Phosphorus from Rich to Poor Patches across Networks

Matthew D. Whiteside, Gijsbert D.A. Werner, Victor E.A. Caldas, Anouk van't Padje, Simon E. Dupin, Bram Elbers, Milenka Bakker, Gregory A.K. Wyatt, Malin Klein, Mark A. Hink, Marten Postma, Bapu Vaitla, Ronald Noë, Thomas S. Shimizu, Stuart A. West, and E. Toby Kiers

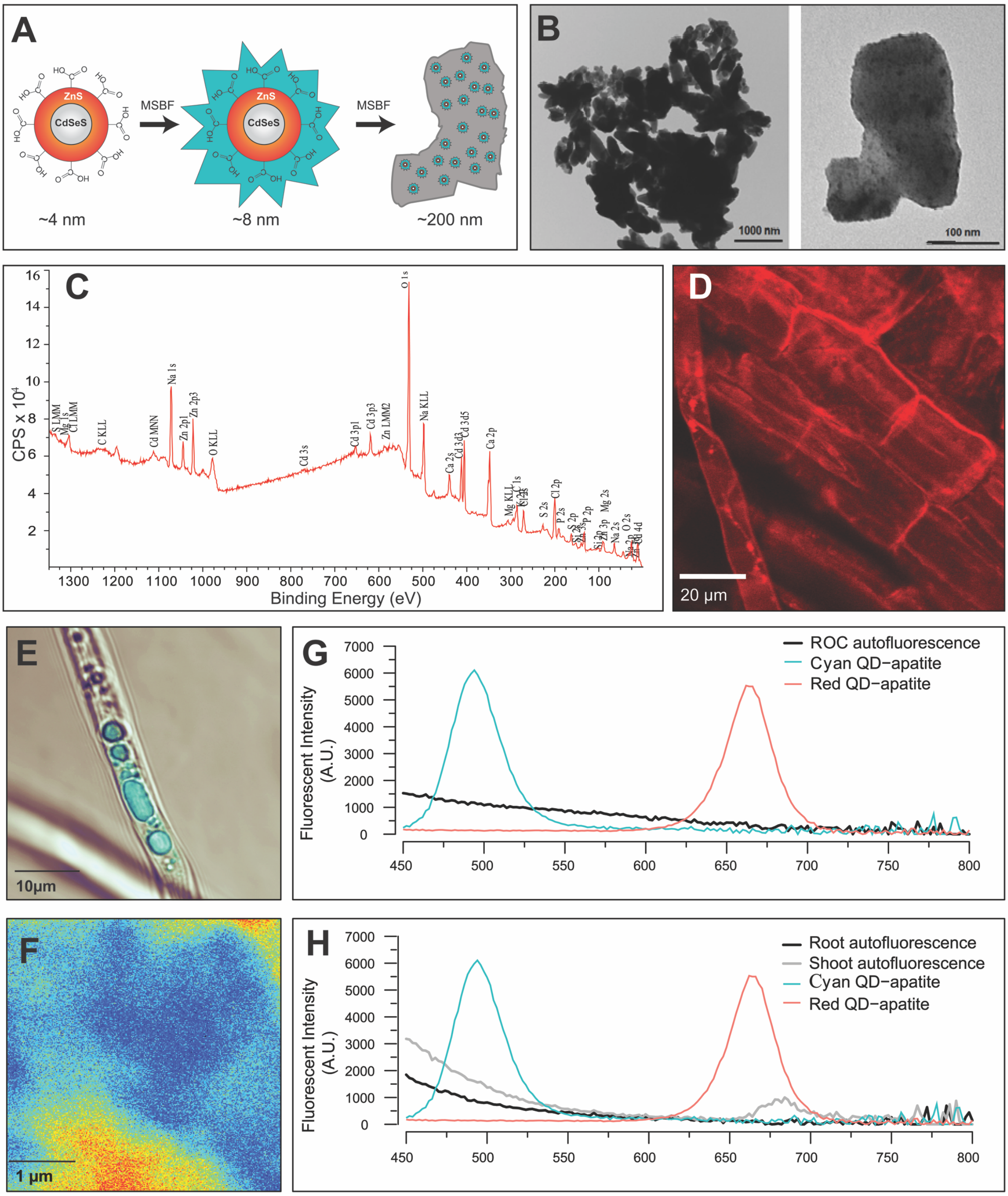

Figure S1. Quantum Dot (QD)-apatite synthesis, structure, imaging and reference emission spectra. Related to Figure 1 and STAR Methods section.

(A) QD-apatite synthesis in which initial reactions with carboxyl-terminated QDs and Modified Simulated Body Fluid (MSBF) result in QD-apatite particles of ~8 nm diameter. These are then re-reacted with modified SBF to form final QD-apatite crystals of ~200 nm diameter. (B) Transmission electron microscope (TEM) images of QD-apatite at low and high resolution fields. Dark and opaque particles in the high-resolution field indicate the presence of quantum dot cores within the apatite coating. (C) X-Ray Photoelectron Spectroscopy analysis to determine the surface structure of the crystalized QD-apatite. Photoelectrons were generated within the X-ray penetration depth of 5-10nm over a 2.0 mm x 0.8 mm surface of dried QD-apatite using a PHI 5701LSci fitted with a

Monochromated Alk 1486.6eV xray source. The detected peaks are used to obtain the composition by integrating the areas under the peaks and applying relative sensitivity factors, assuming a homogeneous layer model. In our QD-apatite samples, the calculated ratio was nmol P:QD = 708:1. Detection limits were approximately 0.05 to 1.0 atomic %. (D) Overlaid confocal scans of red QD-apatite within *M. truncatula* roots colonized with AM fungal hyphae. (E) Uptake of cyan QD-apatite within vacuoles of AM fungal hyphae. (F) False color image of cyan QD-apatite within AM hyphae after RICS analysis in which warmer colors represent a greater concentration of QD-apatite. Image stacks were scanned at 4.096 μm x 4.096 μm with a focal depth of 1.5 μm to ensure that each scan was within the AM fungal hyphae. (G) Reference emission spectra for *in-vitro* cultures and (H) *Medicago truncatula* root and shoot autofluorescence, cyan QD-apatite (488 nm), and red QD-apatite (666 nm). Spectral scans were taken from 450–800 nm at 2 nm intervals using a Bio-Tek plate reader at 325 nm excitation.

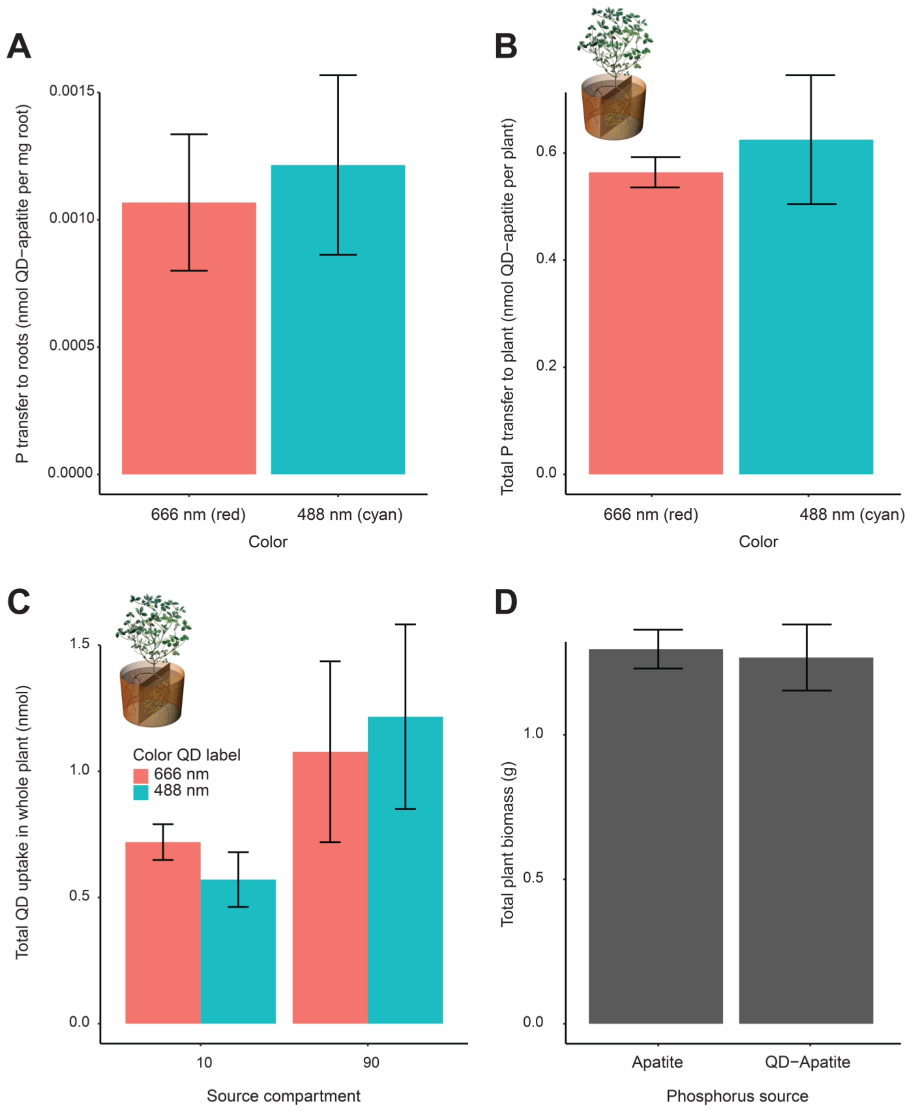

Figure S2. QD-apatite color and toxicity controls. Related to Figure 2 and STAR Methods section

(A) The effect of color on QD-apatite uptake in *in-vitro* root organ cultures colonized by mycorrhizal fungi. When equal amounts of 488 nm cyan and 666 nm red QD-apatite were added to a single fungus-only compartment, we found no indication of differential transfer (Paired t-test t = 0.40, df = 4, P = 0.71, N = 5) Mean transfer ± SE. (B) Total QD-apatite in plant tissue (roots & shoot) of whole plants when split roots were exposed to an equal amount of QD-apatite in a ratio of 50:50. We found no indication of a significant bias toward color (Paired t-test, t = 0.62, df = 19, P = 0.55, N = 20). Mean QD-apatite ± SE. (C) Total QD-apatite in plant tissue (roots & shoot) of mycorrhizal plants when split roots were exposed to an unequal amount of QD-apatite in a ratio of 10:90 to test for color bias. We switched the color treatment, such that 6 plant replicates received Red (666nm) : Cyan (488nm) in a ratio of 10:90. The other 6 plant replicates received a ratio of Cyan (488nm) : Red (666nm) in a ratio of 10:90. We found a marginally significant effect of compartment, with plants accumulating more QD-apatite from 90 compartment, as expected (Wald χ^2 = 3.47, df =1, P = 0.06, N = 12). We found no influence of color on QD-apatite uptake (Wald χ^2 = 0.79, df =1, P = 0.38, N = 12) Mean QD-apatite ± SE. (D) Whole plant toxicity controls using apatite and QD-tagged apatite. We found no significant difference between the apatite and QD-apatite treatments in total plant biomass ($F_{1,23}$ = 0.06, P = 0.81, N = 25). Mean biomass ± SE.

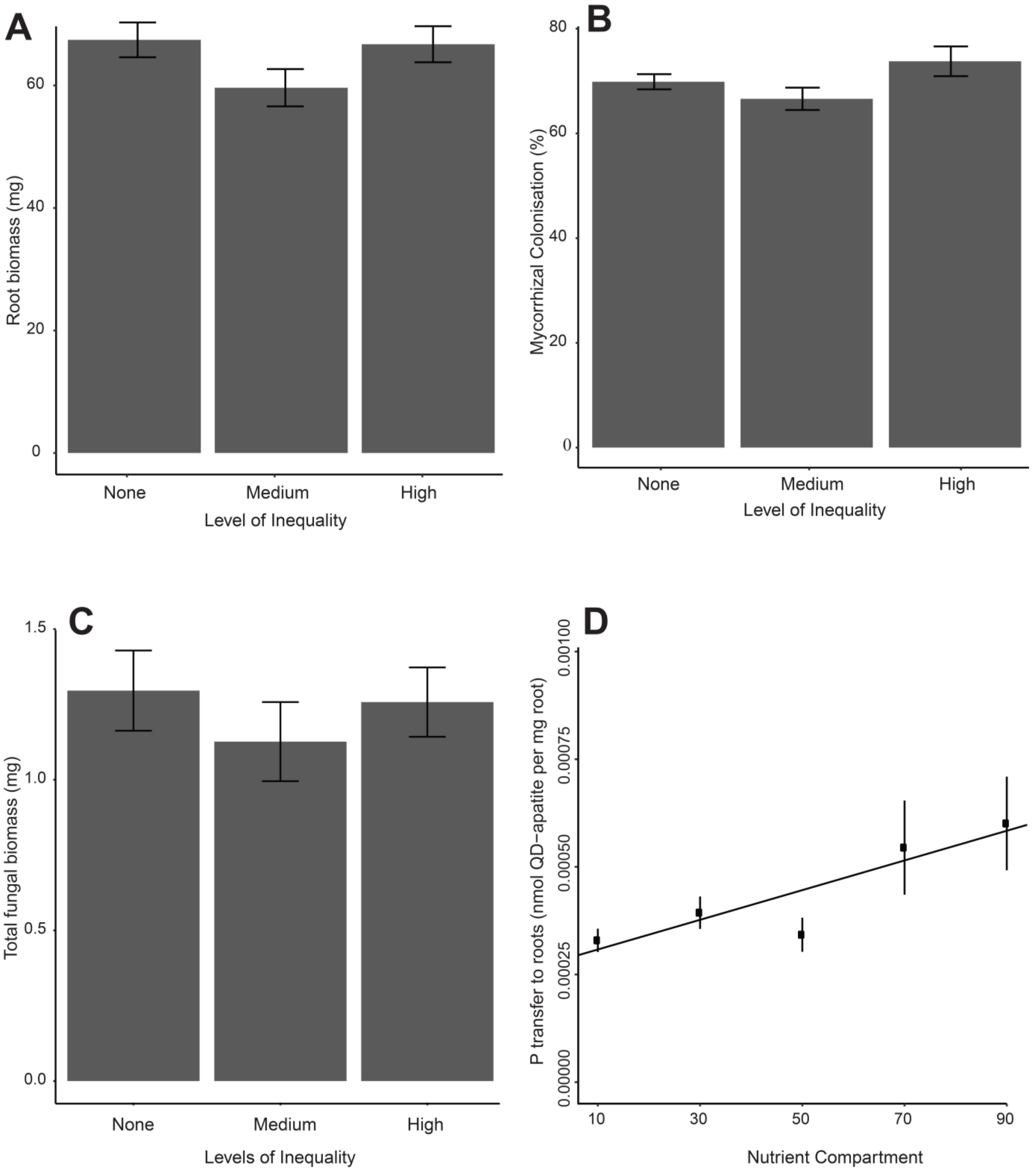

Figure S3. Root and fugal growth. Related to Figure 2 and 3

(A) Biomass of host roots in which fungal network was exposed to three levels of resource inequality. We found that level of inequality treatment had no significant effect on the root biomass of the host ($F_{2,72}$ =2.12, P = 0.1275, N = 75). Mean biomass ± SE. (B) Percent intraradical colonization of host roots in which fungal network was exposed to three levels of resource inequality. We found no significant effect on intradical colonization across inequality treatments ($F_{2,33}$ = 2.62, P = 0.09, N = 36), and no directional pattern. Mean percent colonization ± SE. (C) Total fungal biomass across all three compartments in which fungal network was exposed to three levels of resource inequality. We found no significant effect of inequality on total fungal biomass ($F_{2,56}$ = 0.50, P = 0.61, N =59). Mean biomass ± SE. (D) Phosphorus transfer from a fungus grown in single homogeneous conditions shows a linear relationship with resource level ($F_{1,68}$ = 9.00, $P < 0.01$, N = 70), suggesting that transfer has not reached saturation levels. Data were collected on a three-compartment plate setup in which one compartment was media-free, thereby restricting the network to a single homogenous resource patch varying in P levels (10 = 0.018 nmol, 30 = 0.054 nmol, 50 = 0.09 nmol, 70 = 0.126 nmol, 90 = 0.162 nmol QD-apatite) as in inequality experiment.

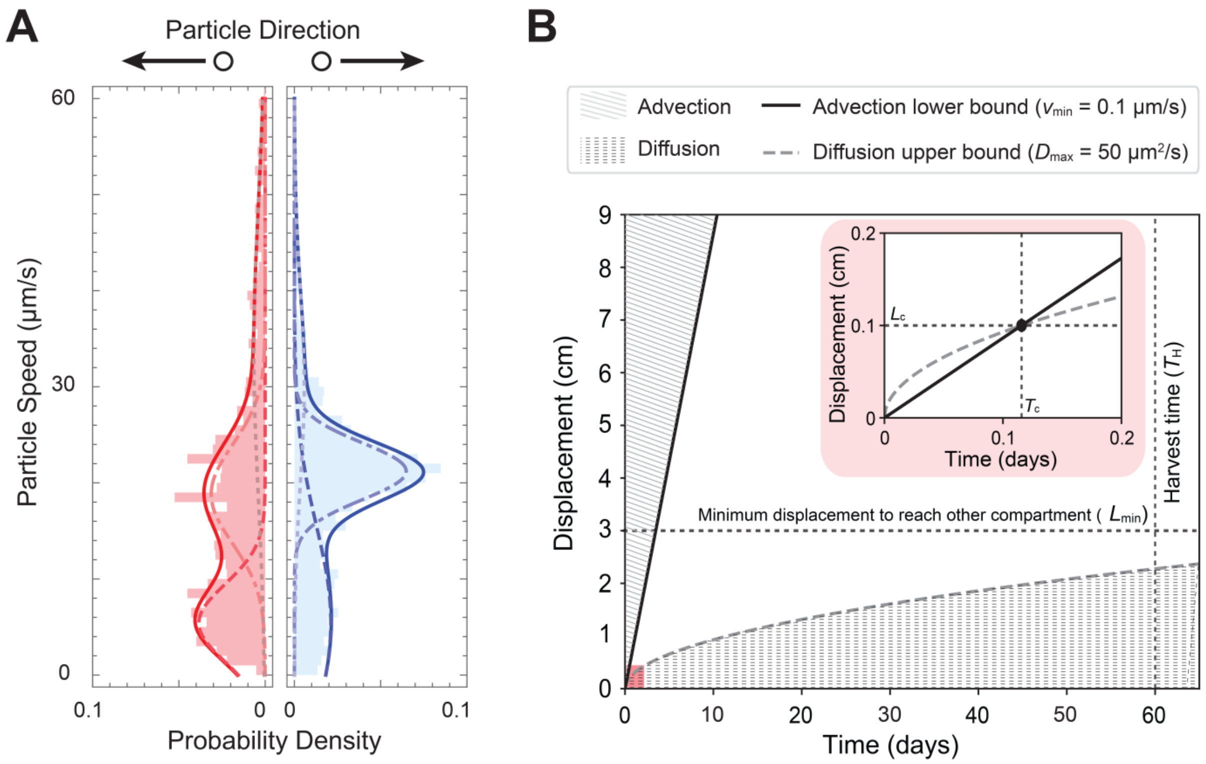

Figure S4. Velocity of cellular contents. Related to Figure 4

(A) Distribution of particle speeds in living fungal network under control conditions from Figure 4B showing multimodal distribution (solid curve) composed of fractions with low (dashed curve), intermediate (dash-dotted curve), and high (dotted curve) mobility. Particle direction (red: leftward, N=1353; blue: rightward, N=1342) was determined by the sign of the velocity vector projection onto a lab-frame axis, where the negative (leftward) direction pointed toward the host root compartment, and the positive (rightward) direction pointed away, and particle speed by the same vector's magnitude. The distribution for each direction was fit to a weighted sum (with weights w_L, w_I, w_H) of three normal distributions (truncated at zero), with parameters (μ_L, σ_L), (μ_I, σ_I), (μ_H, σ_H), where the parameter subscripts L, I, H correspond respectively to the low-, intermediate, and high-mobility fractions. Fit parameter values for the rightward direction: w_L=0.376, w_I=0.481, w_H=0.143, μ_L=5.504, μ_I=21.209, μ_H=30.002, σ_L=9.997, σ_I=2.963, σ_H=10.018. Fit parameter values for the leftward direction were: w_L=0.354, w_I=0.401, w_H=0.245, μ_L=5.416, μ_I=18.822, μ_H=33.715, σ_L=4.032, σ_I=5.122, σ_H=15.094. (B) Expected displacement of phosphorus movement under transport dominated by diffusion (area below dotted line) or advection (area above solid line). Over the time frame ($T_H \approx 60$ days) of the experiment, transport by diffusion alone falls short of the minimum distance to reach the neighboring compartment ($L_{min} \approx 3$ cm). Above the black circle in red inset box represents the cross-over time ($T_c \approx 0.1$ days) and cross-over length ($L_c \approx 0.1$ cm) above which transport by advection dominates over transport by diffusion.

		Poor (%)		Rich (%)	
	Inequality	*Realized*	*Expected*	*Realized*	*Expected*
Transfer	None	51.5 (±4.83)	50	48.5(±4.83)	50
	Medium	44.3 (±5.35)	30	55.7 (±5.35)	70
	High	24.4 (±2.76)	10	75.6 (±2.76)	90
Retention	None	50.8 (±0.63)	50	49.2 (±0.63)	50
	Medium	44.0 (±0.99)	30	56.0 (±0.99)	70
	High	40.9 (±0.75)	10	59.1 (±0.75)	90

Table S1. Realized and expected phosphorus transfer and retention to host root. Related to Figure 2.

Phosphorus transfer and retention (realized and expected) to host root (QD-apatite per mg root ± SE.) by fungus growing in either rich or poor nutrient compartments. The 50:50 treatment provided us with an additional test of the null hypotheses that there was no color effect in either the transfer ($t\ (22) = 0.30$, $p = 0.76$) or retention ($t\ (18) = 1.28$, $p = 0.22$) of QD-apatite.
